# Supplementary material for: Identification and Potential Participation of Lipases in Autophagic Body Degradation in Embryonic Axes of Lupin (Lupinus spp.) Germinating Seeds
Source: Int J Mol Sci. 2023 Dec 20;25(1):90. doi: 10.3390/ijms25010090 (PMC10779169; doi:10.3390/ijms25010090)
Supplement: Supplementary file 1 [file ijms-25-00090-s001.zip › Table S2.pdf]

Table S2. Prediction of subcellular localization of all lipases identified in transcriptomes of white and Andean lupin embryonic axes mapped to narrowleaf lupin (*Lupinus angustifolius* L.) transcriptome (LupAngTanjil_v1.0). The DeepLoc 2.0 software (https://services.healthtech.dtu.dk/services/DeepLoc-2.0/) with a high-quality model was used. A localization is predicted if its probability is above the threshold value (green-filled cells), and the list is sorted by the highest score for vacuolar location. Blue-filled cells indicate the highest score but below the threshold value.

	Gene ID	Protein ID	Name	Cytoplasm	Nucleus	Extra-cellular	Cell membrane	Mitochon-drion	Plastid	Endoplasmic reticulum	**Vacuole**	Golgi apparatus	Peroxisome	Lupin species
			**Probability threshold**	**0.4761**	**0.5014**	**0.6173**	**0.5646**	**0.6220**	**0.6395**	**0.6090**	**0.5848**	**0.6494**	**0.7364**	
1.	XM_019564578.1	XP_019420123.1	lipase-like (LOC109330390)	0.1249	0.0637	0.6849	0.2738	0.0537	0.0589	0.4632	**0.7413**	0.4305	0.0051	white and Andean
2.	XM_019560124.1	XP_019415669.1	sn1-specific diacylglycerol lipase alpha (LOC109327099)	0.3913	0.62264	0.0683	0.5681	0.3879	0.1867	0.5224	**0.7265**	0.6729	0.0083	white and Andean
3.	XM_019578425.1	XP_019433970.1	triacylglycerol lipase 2-like (LOC109340705)	0.1372	0.0666	0.5950	0.2569	0.0253	0.2662	0.4241	**0.7121**	0.3615	0.0235	white and Andean
4.	XM_019570213.1	XP_019425758.1	lipase-like (LOC109334430)	0.1238	0.0633	0.7455	0.2846	0.0734	0.1256	0.4167	**0.7070**	0.4139	0.0128	white and Andean
5.	XM_019604187.1	XP_019459732.1	putative lipase YOR059C (LOC109359495)	0.5098	0.3743	0.1712	0.3106	0.2847	0.2233	0.6089	**0.6995**	0.6764	0.0244	Andean
6.	XM_019580024.1	XP_019435569.1	putative lipase ROG1 (LOC109342050)	0.4751	0.3420	0.1784	0.3196	0.3168	0.2231	0.5423	**0.6956**	0.6792	0.0250	white and Andean
7.	XM_019585936.1	XP_019441481.1	triacylglycerol lipase 1 (LOC109346399)	0.1717	0.1104	0.3269	0.2610	0.0743	0.1614	0.6899	**0.6813**	0.3701	0.0266	white and Andean
8.	XM_019604183.1	XP_019459728.1	GDSL esterase/lipase At2g42990-like (LOC109359493)	0.1070	0.0679	0.5973	0.2432	0.0105	0.1666	0.4311	**0.6752**	0.2104	0.0049	Andean
9.	XM_019565778.1	XP_019421323.1	GDSL esterase/lipase 7-like (LOC109331355)	0.1720	0.0840	0.6110	0.2890	0.0218	0.0991	0.1960	**0.6684**	0.3304	0.0367	Andean
10.	XM_019578399.1	XP_019433944.1	GDSL esterase/lipase At2g42990-like (LOC109340682)	0.1053	0.0639	0.6134	0.2579	0.0107	0.1252	0.4020	**0.6563**	0.2294	0.0056	white and Andean
11.	XM_019583143.1	XP_019438688.1	triacylglycerol lipase 2-like (LOC109344377)	0.0996	0.0750	0.5750	0.2067	0.0248	0.3830	0.4561	**0.6560**	0.2174	0.0211	white and Andean
12.	XM_019568565.1	XP_019424110.1	lipase-like (LOC109333213)	0.4079	0.1664	0.2777	0.1933	0.3235	0.2462	0.7146	**0.6556**	0.7723	0.0595	white and Andean
13.	XM_019568345.1	XP_019423890.1	GDSL esterase/lipase At4g01130-like (LOC109333075)	0.1253	0.0987	0.4851	0.2872	0.0131	0.0569	0.5135	**0.6525**	0.2822	0.0057	white
14.	XM_019603339.1	XP_019458884.1	GDSL esterase/lipase At5g14450 (LOC109358858)	0.1087	0.0985	0.7378	0.1742	0.0168	0.1280	0.3941	**0.6491**	0.1522	0.0055	white and Andean
15.	XM_019566645.1	XP_019422190.1	lipase-like (LOC109331877)	0.4394	0.1665	0.3044	0.2291	0.3315	0.1615	0.6810	**0.6468**	0.7454	0.0281	white and Andean
16.	XM_019575987.1	XP_019431532.1	GDSL esterase/lipase At5g55050-like (LOC109338699)	0.1232	0.0934	0.6040	0.2375	0.0122	0.0618	0.4224	**0.6425**	0.2297	0.0072	white and Andean
17.	XM_019593926.1	XP_019449471.1	GDSL esterase/lipase EXL1-like (LOC109352110)	0.1276	0.0907	0.6176	0.2751	0.0192	0.0835	0.2316	**0.6369**	0.2284	0.0112	white and Andean
18.	XM_019590154.1	XP_019445699.1	GDSL esterase/lipase At5g45960-like (LOC109349384)	0.1051	0.1087	0.5512	0.2341	0.0115	0.1143	0.3511	**0.6302**	0.1974	0.0055	white and Andean
19.	XM_019602401.1	XP_019457946.1	GDSL esterase/lipase At5g22810-like (LOC109358265)	0.1301	0.0966	0.6737	0.2306	0.0147	0.0618	0.4086	**0.6205**	0.1480	0.0043	Andean

20.	XM_019585265.1	XP_019440810.1	GDSL esterase/lipase At1g71691 (LOC109345954)	0.1187	0.0839	0.6802	0.2930	0.0111	0.0466	0.2459	0.6196	0.1618	0.0064	white and Andean
21.	XM_019558678.1	XP_019414223.1	GDSL esterase/lipase EXL3-like (LOC109326040)	0.1235	0.0754	0.5616	0.2526	0.0118	0.0480	0.5097	0.6192	0.2622	0.0051	white and Andean
22.	XM_019574768.1	XP_019430313.1	GDSL esterase/lipase APG-like (LOC109337730)	0.0857	0.0713	0.7163	0.2529	0.0099	0.1867	0.4058	0.6156	0.1377	0.0076	Andean
23.	XM_019603544.1	XP_019459089.1	GDSL esterase/lipase 5-like (LOC109359036)	0.0988	0.0939	0.5668	0.2812	0.0168	0.0886	0.3382	0.6152	0.2027	0.0060	white and Andean
24.	XM_019573752.1	XP_019429297.1	GDSL esterase/lipase APG-like (LOC109336918)	0.0973	0.0867	0.6546	0.2086	0.0087	0.1593	0.3978	0.6094	0.1387	0.0056	white and Andean
25.	XM_019590739.1<br>XM_019590740.1	XP_019446284.1<br>XP_019446285.1	lipase 3-like (LOC109349776)	0.3293	0.1463	0.1357	0.2716	0.2600	0.1613	0.6459	0.6063	0.6241	0.1617	white<br>Andean
26.	XM_019598362.1	XP_019453907.1	GDSL esterase/lipase At5g22810-like (LOC109355290)	0.1068	0.0656	0.7004	0.1867	0.0132	0.1135	0.3575	0.6060	0.1499	0.0088	Andean
27.	XM_019580064.1	XP_019435609.1	GDSL esterase/lipase At5g55050-like (LOC109342072)	0.1154	0.1049	0.5881	0.2344	0.0153	0.0821	0.3895	0.6032	0.2036	0.0039	white and Andean
28.	XM_019574906.1	XP_019430451.1	monoacylglycerol lipase-like (LOC109337835)	0.1607	0.1250	0.4608	0.2318	0.0163	0.1849	0.2884	0.6006	0.2001	0.0119	Andean
29.	XM_019597714.1	XP_019453259.1	GDSL esterase/lipase EXL1-like (LOC109354906)	0.1607	0.1250	0.4608	0.2318	0.0163	0.1849	0.2884	0.6006	0.2001	0.0119	white and Andean
30.	XM_019577313.1	XP_019432858.1	GDSL esterase/lipase At5g18430-like (LOC109339796)	0.1155	0.1176	0.6524	0.1599	0.0163	0.2249	0.3856	0.5976	0.1010	0.0071	white and Andean
31.	XM_019584670.1	XP_019440215.1	GDSL esterase/lipase At5g45910-like (LOC109345584)	0.1155	0.1176	0.6524	0.1599	0.0163	0.2249	0.3856	0.5976	0.1010	0.0071	white
32.	XM_019606974.1	XP_019462519.1	GDSL esterase/lipase At5g45670-like (LOC109361506)	0.1745	0.1344	0.6873	0.2629	0.0219	0.0575	0.3940	0.5970	0.1796	0.0034	white and Andean
33.	XM_019560336.1	XP_019415881.1	putative lipase YDL109C (LOC109327267)	0.5177	0.2787	0.1145	0.5016	0.2160	0.3013	0.5523	0.5947	0.4328	0.0594	white and Andean
34.	XM_019603098.1	XP_019458643.1	GDSL esterase/lipase At2g42990-like (LOC109358701)	0.1742	0.1030	0.6854	0.1671	0.0073	0.1844	0.3105	0.5943	0.1800	0.0192	white and Andean
35.	XM_019568357.1	XP_019423902.1	GDSL esterase/lipase At4g01130-like (LOC109333086)	0.0970	0.0669	0.6555	0.2113	0.0174	0.0771	0.3975	0.5898	0.1388	0.0032	white
36.	XM_019598852.1	XP_019454397.1	GDSL esterase/lipase At1g71250-like (LOC109355611)	0.1263	0.1557	0.6132	0.2149	0.0178	0.1169	0.3113	0.5887	0.1588	0.0057	white and Andean
37.	XM_019605087.1	XP_019460632.1	GDSL esterase/lipase At1g71250-like (LOC109360298)	0.1523	0.1259	0.5000	0.3009	0.0281	0.1422	0.3990	0.5884	0.3172	0.0111	white and Andean
38.	XM_019581118.1	XP_019436663.1	probable lipase C1672.09 (LOC109343013)	0.2891	0.1719	0.1389	0.3496	0.2474	0.1869	0.6562	0.5852	0.6347	0.1633	white and Andean
39.	XM_019600195.1	XP_019455740.1	GDSL esterase/lipase At5g03610-like (LOC109356708)	0.1017	0.1107	0.5988	0.2107	0.0183	0.0686	0.3174	0.5843	0.1577	0.0066	white and Andean
40.	XM_019609193.1	XP_019464738.1	GDSL esterase/lipase At3g48460-like (LOC109363031)	0.1021	0.1036	0.6847	0.2375	0.0247	0.2707	0.3062	0.5831	0.1404	0.0044	Andean
41.	XM_019568366.1	XP_019423911.1	GDSL esterase/lipase At4g01130-like (LOC109333086)	0.0943	0.0634	0.6706	0.2144	0.0162	0.0696	0.3955	0.5824	0.1360	0.0033	Andean
42.	XM_019561187.1	XP_019416732.1	GDSL esterase/lipase EXL3-like (LOC109327976)	0.1095	0.0750	0.5532	0.2849	0.0167	0.0647	0.3631	0.5803	0.1897	0.0023	Andean

43.	XM_019597715.1	XP_019453260.1	GDSL esterase/lipase EXL3-like (LOC109354907)	0.0996	0.0752	0.6073	0.2578	0.0144	0.1184	0.2916	0.5787	0.1906	0.0041	white and Andean
44.	XM_019570663.1	XP_019426208.1	putative lipase C4A8.10 (LOC109334740)	0.4149	0.4287	0.1440	0.2787	0.2813	0.2795	0.4887	0.5761	0.4958	0.0648	Andean
45.	XM_019563836.1	XP_019419381.1	GDSL esterase/lipase At1g29660-like (LOC109329930)	0.1054	0.0582	0.6445	0.2074	0.0214	0.0656	0.3505	0.5760	0.1286	0.0042	white
46.	XM_019568564.1	XP_019424109.1	lipase-like (LOC109333212)	0.3310	0.1018	0.4708	0.1879	0.2300	0.1466	0.6539	0.5750	0.6744	0.0199	white and Andean
47.	XM_019589143.1	XP_019444688.1	GDSL esterase/lipase At5g45670-like (LOC109348632)	0.1504	0.1431	0.6795	0.2890	0.0237	0.1012	0.3445	0.5744	0.1579	0.0038	white and Andean
48.	XM_019597340.1	XP_019452885.1	GDSL esterase/lipase At3g27950 (LOC109354679)	0.0796	0.0833	0.5909	0.1808	0.0236	0.1612	0.2695	0.5741	0.1660	0.0150	white and Andean
49.	XM_019583157.1	XP_019438702.1	GDSL esterase/lipase At5g14450-like (LOC109344386)	0.0945	0.1334	0.6650	0.2472	0.0320	0.0588	0.2669	0.5741	0.1726	0.0042	Andean
50.	XM_019570638.1	XP_019426183.1	GDSL esterase/lipase At1g28600-like (LOC109334728)	0.1246	0.0831	0.6486	0.2022	0.0138	0.1643	0.4517	0.5738	0.1223	0.0127	white and Andean
51.	XM_019580927.1	XP_019436472.1	GDSL esterase/lipase APG-like (LOC109342884)	0.0842	0.0747	0.7277	0.2590	0.0127	0.2574	0.3531	0.5729	0.1089	0.0048	white and Andean
52.	XM_019563456.1	XP_019419001.1	GDSL esterase/lipase LTL1-like (LOC109329702)	0.1635	0.0912	0.5201	0.2274	0.0113	0.1889	0.4198	0.5728	0.1683	0.0061	white and Andean
53.	XM_019603110.1	XP_019458655.1	lipase member M-like (LOC109358709)	0.2857	0.1649	0.1258	0.3495	0.2463	0.1516	0.6490	0.5707	0.6287	0.1344	white and Andean
54.	XM_019578333.1	XP_019433878.1	GDSL esterase/lipase EXL1-like (LOC109340609)	0.0971	0.0592	0.7441	0.2190	0.0152	0.2111	0.2132	0.5600	0.1469	0.0080	Andean
55.	XM_019574983.1	XP_019430528.1	GDSL esterase/lipase At1g29660-like (LOC109337900)	0.0916	0.0648	0.7231	0.1839	0.0166	0.0765	0.3781	0.5547	0.1296	0.0048	white
56.	XM_019605956.1	XP_019461501.1	GDSL esterase/lipase At2g30310-like (LOC109360817)	0.1674	0.1461	0.5968	0.2081	0.0151	0.1058	0.3789	0.5524	0.1707	0.0036	white
57.	XM_019584665.1	XP_019440210.1	GDSL esterase/lipase At5g45910-like (LOC109345581)	0.0940	0.0851	0.7228	0.1867	0.0200	0.1590	0.3395	0.5497	0.1130	0.0033	Andean
58.	XM_019601223.1	XP_019456768.1	GDSL esterase/lipase At2g04570-like (LOC109357323)	0.1219	0.1127	0.4744	0.2163	0.0141	0.3469	0.2758	0.5474	0.1231	0.0117	white and Andean
59.	XM_019586660.1	XP_019442205.1	GDSL esterase/lipase At3g27950-like (LOC109346920)	0.0869	0.0794	0.7865	0.1949	0.0311	0.1124	0.3608	0.5451	0.1637	0.0039	white and Andean
60.	XM_019606453.1	XP_019461998.1	GDSL esterase/lipase At3g26430 (LOC109361126)	0.1117	0.0898	0.5876	0.2296	0.0192	0.3077	0.4904	0.5440	0.2408	0.0054	white and Andean
61.	XM_019604256.1	XP_019459801.1	GDSL esterase/lipase At5g03610-like (LOC109359550)	0.1418	0.0968	0.5890	0.1631	0.0151	0.0774	0.4261	0.5363	0.1678	0.0047	Andean
62.	XM_019578335.1	XP_019433880.1	GDSL esterase/lipase EXL1-like (LOC109340612)	0.1180	0.0939	0.5863	0.2083	0.0144	0.1856	0.2300	0.5289	0.1240	0.0083	white and Andean
63.	XM_019606343.1	XP_019461888.1	GDSL esterase/lipase At5g37690 (LOC109361044)	0.1240	0.1090	0.7246	0.1863	0.0215	0.1348	0.3559	0.5274	0.1168	0.0089	white and Andean
64.	XM_019569235.1	XP_019424780.1	GDSL esterase/lipase At1g33811 (LOC109333690)	0.1111	0.0690	0.7143	0.2712	0.0157	0.1500	0.2717	0.5252	0.1583	0.0084	white and Andean
65.	XM_019559601.1	XP_019415146.1	monoglyceride lipase-like (LOC109326794)	0.3360	0.2818	0.0771	0.2577	0.2003	0.3708	0.8008	0.5237	0.5365	0.2093	white and Andean
66.	XM_019563839.1	XP_019419384.1	GDSL esterase/lipase At1g29670-like (LOC109329931)	0.0950	0.1040	0.7550	0.2422	0.0134	0.0754	0.2375	0.5182	0.0928	0.0034	white and Andean

67.	XM_019608640.1	XP_019464185.1	lipase-like PAD4 (LOC109362676)	0.6540	0.4268	0.1075	0.1444	0.2189	0.2926	0.2933	0.5170	0.4645	0.1761	white and Andean
68.	XM_019587276.1	XP_019442821.1	GDSL esterase/lipase At5g33370-like (LOC109347427)	0.0969	0.0888	0.6887	0.2467	0.0172	0.1158	0.3305	0.5127	0.1916	0.0028	white
69.	XM_019568009.1	XP_019423554.1	GDSL esterase/lipase 5-like (LOC109332879)	0.1058	0.0961	0.5731	0.1957	0.0183	0.1768	0.3594	0.5100	0.0980	0.0070	white and Andean
70.	XM_019566899.1	XP_019422444.1	GDSL esterase/lipase At1g09390-like (LOC109332072)	0.1223	0.0996	0.5903	0.2005	0.0111	0.1700	0.3801	0.5094	0.1165	0.0091	white
71.	XM_019566904.1	XP_019422449.1	GDSL esterase/lipase At1g09390-like (LOC109332072)	0.1223	0.0996	0.5903	0.2005	0.0111	0.1700	0.3801	0.5094	0.1165	0.0091	Andean
72	XM_019592904.1	XP_019448449.1	GDSL esterase/lipase At5g33370-like (LOC109351399)	0.1171	0.0696	0.6058	0.2136	0.0131	0.2282	0.4601	0.4984	0.1644	0.0058	white and Andean
73.	XM_019578371.1	XP_019433916.1	GDSL esterase/lipase At1g71250-like (LOC109340651)	0.1521	0.1136	0.5826	0.2642	0.0148	0.0553	0.3346	0.4933	0.1493	0.0100	Andean
74.	XM_019558709.1	XP_019414254.1	lipase-like PAD4 (LOC109326062)	0.6890	0.4740	0.1101	0.0940	0.2544	0.1910	0.3015	0.4893	0.4663	0.3869	white and Andean
75.	XM_019586935.1	XP_019442480.1	GDSL esterase/lipase At5g03610-like (LOC109347229)	0.0834	0.0957	0.6297	0.1565	0.0190	0.1696	0.3619	0.4884	0.0978	0.0033	white and Andean
76.	XM_019562443.1	XP_019417988.1	GDSL esterase/lipase At1g54790 (LOC109328837)	0.1160	0.0756	0.6198	0.2009	0.0191	0.3269	0.3690	0.4841	0.1092	0.0044	white and Andean
77.	XM_019563457.1	XP_019419002.1	GDSL esterase/lipase At5g33370-like (LOC109329703)	0.1212	0.0639	0.6826	0.3159	0.0145	0.0848	0.3368	0.4791	0.1752	0.0021	white and Andean
78.	XM_019562395.1	XP_019417940.1	monoacylglycerol lipase-like (LOC109328777)	0.3415	0.2848	0.1194	0.3226	0.2189	0.4705	0.7592	0.4786	0.5001	0.2768	white and Andean
79.	XM_019567499.1	XP_019423044.1	GDSL esterase/lipase At2g03980-like (LOC109332513)	0.0952	0.1053	0.6235	0.2393	0.0112	0.2286	0.2003	0.4726	0.1207	0.0102	Andean
80.	XM_019568922.1	XP_019424467.1	GDSL esterase/lipase At2g04570-like (LOC109333435)	0.0952	0.1053	0.6235	0.2393	0.0112	0.2286	0.2003	0.4726	0.1207	0.0102	white and Andean
81.	XM_019570437.1	XP_019425982.1	GDSL esterase/lipase At5g45910-like (LOC109334589)	0.0955	0.0837	0.7125	0.2368	0.0223	0.3743	0.2581	0.4710	0.0976	0.0176	Andean
82.	XM_019608191.1	XP_019463736.1	GDSL esterase/lipase At3g48460-like (LOC109362412)	0.0960	0.1170	0.6303	0.1987	0.0202	0.2842	0.2958	0.4702	0.1098	0.0046	white and Andean
83.	XM_019607055.1	XP_019462600.1	monoacylglycerol lipase ABHD6-like (LOC109361563)	0.4359	0.2383	0.0981	0.5731	0.4030	0.2756	0.7035	0.4679	0.2393	0.0461	white
84.	XM_019565795.1	XP_019421340.1	monoacylglycerol lipase ABHD6-like (LOC109331365)	0.3903	0.2329	0.0651	0.4935	0.5056	0.2111	0.7683	0.4591	0.3554	0.0282	white and Andean
85.	XM_019606808.1	XP_019462353.1	GDSL esterase/lipase 1-like (LOC109361345)	0.1288	0.1528	0.6377	0.1256	0.0186	0.1331	0.3325	0.4582	0.1411	0.0060	Andean
86.	XM_019566757.1	XP_019422302.1	GDSL esterase/lipase At4g10955-like (LOC109331945)	0.6453	0.3422	0.1508	0.1146	0.2286	0.4379	0.4634	0.4500	0.6277	0.0748	white and Andean
87.	XM_019589079.1	XP_019444624.1	GDSL esterase/lipase At4g10955-like (LOC109348588)	0.6173	0.3836	0.1167	0.1469	0.2232	0.3508	0.4190	0.4470	0.5623	0.0355	white and Andean
88.	XM_019571265.1	XP_019426810.1	GDSL esterase/lipase At5g03610-like (LOC109335167)	0.0992	0.1335	0.5656	0.1813	0.0178	0.1301	0.2458	0.4241	0.0970	0.0047	white and Andean
89.	XM_019572916.1	XP_019428461.1	GDSL esterase/lipase At5g33370-like (LOC109336364)	0.1295	0.0960	0.6964	0.3444	0.0181	0.1166	0.3356	0.4167	0.1457	0.0035	Andean

90.	XM_019567821.1	XP_019423366.1	GDSL esterase/lipase At4g10955-like (LOC109332786)	0.5925	0.3438	0.1492	0.1257	0.2443	0.4819	0.4027	0.4083	0.5844	0.0771	Andean
91.	XM_019606596.1	XP_019462141.1	monoacylglycerol lipase ABHD6-like (LOC109361216)	0.3975	0.2823	0.1087	0.5710	0.3189	0.2704	0.5286	0.4081	0.1002	0.0368	white and Andean
92.	XM_019586384.1	XP_019441929.1	sn1-specific diacylglycerol lipase beta (LOC109346684)	0.0759	0.0931	0.0770	0.2424	0.6519	0.7260	0.2014	0.3905	0.3564	0.2010	white and Andean
93.	XM_019601877.1	XP_019457422.1	GDSL esterase/lipase 1-like (LOC109357820)	0.1064	0.1264	0.6373	0.1709	0.0153	0.1646	0.2641	0.3902	0.1130	0.0149	white and Andean
94.	XM_019563453.1	XP_019418998.1	GDSL esterase/lipase At5g33370-like (LOC109329700)	0.1492	0.1473	0.5994	0.2676	0.0217	0.2312	0.4421	0.3897	0.1098	0.0049	Andean
95.	XM_019563390.1	XP_019418935.1	monoacylglycerol lipase ABHD6-like (LOC109329668)	0.3399	0.2284	0.0783	0.4049	0.3401	0.4195	0.5025	0.3888	0.1716	0.0847	white and Andean
96.	XM_019597471.1	XP_019453016.1	GDSL esterase/lipase At5g33370-like (LOC109354764)	0.0854	0.0605	0.7395	0.2656	0.0193	0.2554	0.3080	0.3847	0.1172	0.0044	white and Andean
97.	XM_019607054.1	XP_019462599.1	monoacylglycerol lipase ABHD6-like (LOC109361563)	0.4174	0.2551	0.0836	0.4160	0.3903	0.1917	0.7951	0.3801	0.1429	0.0853	Andean
98.	XM_019579595.1	XP_019435140.1	GDSL esterase/lipase At4g10955-like (LOC109341649)	0.5077	0.2582	0.1841	0.2128	0.2294	0.3736	0.2953	0.3464	0.4444	0.1770	white and Andean
99.	XM_019565794.1	XP_019421339.1	monoacylglycerol lipase ABHD6-like (LOC109331364)	0.3716	0.2489	0.0934	0.4767	0.4662	0.5820	0.6299	0.3395	0.1711	0.1070	white and Andean
100.	XM_019585183.1	XP_019440728.1	GDSL esterase/lipase At4g10955 (LOC109345911)	0.4734	0.2726	0.1215	0.2042	0.2266	0.3683	0.3545	0.3285	0.4201	0.0971	white and Andean
101.	XM_019599596.1	XP_019455141.1	GDSL esterase/lipase At2g03980-like (LOC109356271)	0.1245	0.1003	0.7277	0.2006	0.0301	0.1368	0.3421	0.2715	0.0507	0.0079	Andean
102.	XM_019558759.1	XP_019414304.1	GDSL esterase/lipase CPRD49-like (LOC109326099)	0.6908	0.5051	0.1221	0.1211	0.1783	0.1433	0.2359	0.2675	0.1444	0.3008	white
103.	XM_019558758.1	XP_019414303.1	GDSL esterase/lipase CPRD49-like (LOC109326099)	0.6889	0.5096	0.1191	0.1184	0.1744	0.1449	0.2349	0.2646	0.1442	0.3109	Andean
104.	XM_019594325.1	XP_019449870.1	GDSL esterase/lipase CPRD49-like (LOC109352366)	0.6964	0.5492	0.0951	0.1310	0.1652	0.1485	0.2168	0.2382	0.1442	0.2539	white and Andean
105.	XM_019574165.1	XP_019429710.1	triacylglycerol lipase SDP1-like (LOC109337240)	0.1951	0.2585	0.0451	0.2253	0.4544	0.1814	0.6865	0.2260	0.2823	0.6608	white and Andean
106.	XM_019579446.1	XP_019434991.1	triacylglycerol lipase SDP1-like (LOC109341517)	0.1951	0.2585	0.0451	0.2253	0.4544	0.1814	0.6865	0.2260	0.2823	0.6608	white and Andean
107.	XM_019577317.1	XP_019432862.1	GDSL esterase/lipase At5g33370-like (LOC109339800)	0.2068	0.2479	0.6369	0.5579	0.0579	0.1783	0.3797	0.2227	0.0927	0.1761	Andean
108.	XM_019607168.1	XP_019462713.1	GDSL esterase/lipase CPRD49-like (LOC109361650)	0.6620	0.5318	0.0968	0.1067	0.1371	0.1764	0.1674	0.2175	0.1858	0.1671	Andean
109.	XM_019588469.1	XP_019444014.1	triacylglycerol lipase SDP1-like (LOC109348192)	0.2458	0.2684	0.0505	0.2260	0.5387	0.1973	0.6460	0.2162	0.3208	0.7435	white and Andean
110.	XM_019567818.1	XP_019423363.1	GDSL esterase/lipase At4g10955-like (LOC109332786)	0.4008	0.2823	0.1915	0.1154	0.2627	0.7597	0.3221	0.2150	0.2084	0.0347	white and Andean
111.	XM_019570500.1	XP_019426045.1	GDSL esterase/lipase At5g45920-like (LOC109334624)	0.6921	0.5050	0.0627	0.0863	0.1546	0.0840	0.1058	0.1973	0.1809	0.2307	white and Andean
112.	XM_019560520.1	XP_019416065.1	GDSL esterase/lipase At5g45920-like (LOC109327394)	0.5612	0.4240	0.1136	0.0879	0.2198	0.2114	0.1109	0.1477	0.0859	0.4696	white and Andean

113.	XM_019595094.1	XP_019450639.1	GDSL esterase/lipase At5g03810-like (LOC109352908)	0.3702	0.3767	0.7247	0.3323	0.0632	0.2770	0.2085	0.1351	0.1329	0.1174	white and Andean
114.	XM_019608481.1	XP_019464026.1	GDSL esterase/lipase At5g62930 (LOC109362582)	0.6981	0.4529	0.1087	0.0922	0.1483	0.1192	0.1605	0.1242	0.1433	0.1921	white and Andean
115.	XM_019608274.1	XP_019463819.1	putative lipase C4A8.10 (LOC109362465)	0.1751	0.1502	0.0295	0.1669	0.2054	0.9681	0.1051	0.0980	0.0996	0.0728	white and Andean
116.	XM_019557749.1	XP_019413294.1	GDSL esterase/lipase CPRD49-like (LOC109325402)	0.1697	0.1117	0.0440	0.0799	0.0824	0.9920	0.0529	0.0749	0.0458	0.0459	Andean
117.	XM_019599793.1	XP_019455338.1	GDSL esterase/lipase 7-like (LOC109356478)	0.4547	0.3306	0.4613	0.1430	0.1448	0.2274	0.2467	0.0702	0.0422	0.0354	Andean
118.	XM_019557757.1	XP_019413302.1	GDSL esterase/lipase At2g38180-like (LOC109325407)	0.1750	0.1404	0.0257	0.0748	0.1058	0.9880	0.0380	0.0690	0.0313	0.0641	white and Andean
119.	XM_019557744.1	XP_019413289.1	GDSL esterase/lipase CPRD49-like (LOC109325402)	0.1656	0.1211	0.0489	0.0937	0.0977	0.9915	0.0516	0.0658	0.0382	0.0566	white
120.	XM_019589071.1	XP_019444616.1	putative esterase/lipase HI_0193 (LOC109348582)	0.3137	0.1835	0.0341	0.0444	0.7467	0.5979	0.0989	0.0657	0.0492	0.5351	Andean
121.	XM_019599598.1	XP_019455143.1	GDSL esterase/lipase At5g55050-like (LOC109356274)	0.5490	0.2248	0.3717	0.1755	0.0808	0.1094	0.3537	0.0529	0.0309	0.0636	Andean
122.	XM_019589069.1	XP_019444614.1	putative esterase/lipase HI_0193 (LOC109348582)	0.1040	0.0976	0.0145	0.0533	0.2134	0.9927	0.0246	0.0428	0.0643	0.0855	white and Andean
